# Microstructure and corrosion resistance of high entropy alloy (AlNiCoCrFe) coatings prepared by TIG process

**DOI:** 10.1016/j.heliyon.2024.e41062

**Published:** 2024-12-09

**Authors:** Mahmoud Ardeshir, Mardali Yousefpour, Seyad Mohammad Sadegh Nourbabksh, Mansoor Bozorg

**Affiliations:** a Faculty of Materials and Metallurgical Engineering, Semnan University, Semnan, 35131-19111, Iran; b Faculty of Chemical and Materials Engineering, Sharood University of Technology, Sharood, 3619995161, Iran

**Keywords:** High entropy alloy, Gas tungsten arc, Hard facing coating, FCC solid solution, Electrochemical measurements

## Abstract

Corrosion resistance, hardness and other mechanical properties of high entropy alloys are enhanced due to the addition of the proper elements. In this study, an equimolar powder mixture of AlNiCoCrFe was prepared as a coating material on plain carbon steel. It was produced by gas tungsten arc welding with the electrical currents of 90, 110 and 130 A. Experimental results demonstrated that at the electrical current of 110 A, the AlNiCoCrFe layer contained two-phase structures of BCC and FCC. The layer exhibited improved hardness from about 180 to 650 HVN. The potentiodynamic polarisation and electrochemical impedance spectroscopy of the AlNiCoCrFe alloys, obtained in 1M HCl solutions, showed that corrosion resistance of steel substrate could be considerably improved by preparing an AlNiCoCrFe coating on its surface. Polarisation resistance increased from 62.5 to 834 Ωcm^2^. Furthermore, electrochemical measurements revealed that optimum corrosion resistance were obtained at 110 A electrical current.

## Highlights

• TIG process was utilised to produce a hard high entropy alloy layer on carbon steel.
• The volume of pool on the surface has a critical influence on determination of phase.
• The optimum current (110 A) was obtained to form two phases of FCC and BCC.

## Graphical abstract

**Figure:**
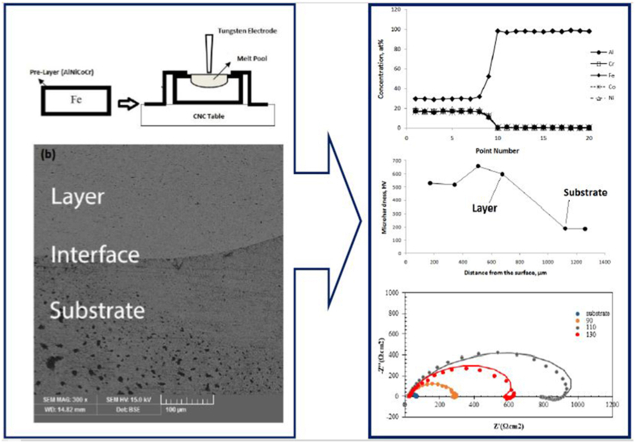

## 1 Introduction

Recently, novel materials called high entropy alloys (HEAs) were developed by Yeh et al. [[1], [2], [3], [4], [5]]. Two definitions [6,7] were provided for HEAs. The basis of the first definition is the chemical composition and the basis of the second one is their configurational entropy. In the first definition, HEAs must contain at least five elements, where each of their concentrations varies between 5 and 35 %. In the second definition, the configurational entropies of HEAs must exceed 1.5R (R is gas constant), regardless [8] of whether they are single-phase or multiphase at room temperature. High hardness, good wear properties and excellent corrosion resistance are observed properties of high entropy materials [[9], [10], [11], [12], [13]]. Casting [14], powder metallurgy [15], cladding [16], spraying [17], mechanical alloying [18] and welding procedures [19] are the current methods used to produce the HEA systems that have been routinely considered in different studies. Among these, AlNiCoCrFe coatings produced via the tungsten inert gas (TIG) process represent a promising avenue for enhancing the performance of materials in corrosive environments. The TIG process, known for its ability to produce high quality welds and coatings, allows for precise control over the microstructural characteristics of the deposited alloy [19]. However, there is abundant literature that notes that HEAs contain multiple elements as a bulk material. Furthermore, the dimensions and form of bulk ingots made using pointed methods were limited. Hence, some scientists have been trying to probe the two-dimensional production of a layer of high entropy alloy on cheap metallic substrates [[20], [21], [22]]. One of the current methods is laser processing [[23], [24], [25]] and another current and low-cost methods of surface hardening and alloying is the use of tungsten inert gas (TIG), which can easily provide the strong and sound bond between layer and substrate [26,27]. The layer preparation of multi element alloys has seldom been considered. In situ synthesized high-entropy alloy coatings have been focused with the production approach of the multicomponent system with the TIG procedure [28]. For instance, Jie-Hao Chen et al. [19] have fabricated a HEA layer on steel substrate using the tungsten inert gas process in which they used Ni, Co, Cr, Mo and Al. In another experiment, Y.C. Lin [28] had successfully blended two powder mixtures of NiCrAlCoW and NiCrAlCoSi as an initial material. The coating was produced by TIG on plain carbon steel substrate to produce an in situ synthesized layer.

The TIG process is a low-cost method for producing HEA coatings, making it a more economical alternative to traditional coating methods. The importance of the HEA coatings in recent research lies in the evaluation of properties and behaviour of this kind of material in different conditions like harsh environments, especially in corrosive solutions. Furthermore, there is still a need to understand the effects of processing parameters, such as electrical current, on the microstructure, phases, and properties of HEA coatings produced by the TIG process.

The AlNiCoCrFe alloy has unique combination of properties, including high corrosion resistance, good wear resistance, and high hardness. This alloy has been shown to exhibit a single-phase face-centered cubic (FCC) structure, which is beneficial for achieving high corrosion resistance and mechanical properties.

Qiu et al. [29] investigated the electrochemical properties of Al2CrFeCoxCuNiTi HEAs coatings prepared on Q235 steel by laser cladding. The results showed that the coating with x = 2 showed better corrosion resistance than 304 stainless steel in the H_2_SO_4_ solution. The effect of Al on corrosion properties of AlxFeCoNiCuCr HEAs coatings prepared on AISI 1045 steel was reported by Ye et al. [8]. The results indicated that AlxFeCoNiCrTi coatings prepared by laser cladding are better than 314L stainless steel in corrosion resistance, in which Al1.8FeCoNiCrTi coatings serve the best.

This study aims to investigate the possibility of the production of coatings containing high entropy alloys on plain carbon steel substrate using the tungsten inert gas process under controlled conditions to achieve a suitable coating and improve surface properties and increase the corrosion resistance of the substrate. In this way, the same equimolar powder mixtures were used on AISI 1050 medium carbon steel. The effect of electrical current on the microstructure, phases and properties of the coatings during the TIG process has not yet been investigated.

## 2 Materials and methods

The substrate samples were AISI 1050 carbon steel with thickness of 20 mm, length of 400 mm and width of 20 mm. the samples were cut from a slab. The elements Aluminium, Cobalt, Chromium and Nickel were used as the principal elements in the powdered form. The powders were with purity higher than 99.5 % and particle size of approximate 60 to 120 μm. These elements were blended to prepare a base material for the layer. After blending for 8 h, 5 wt% polyvinyl alcohol was added to the mixtures as a binder. The ball milling machine was used to blend and mix the powders with 250 r/min speed. Then, a uniformed mixture was applied as a pre-layer on the surface of 20 × 40 mm^2^ of AISI 1050 carbon steel. The thickness of the pre-layer was 1 mm. In the next step, samples were heated to 70 °C for 4 h to dry, so that the moisture would evaporate. Finally, surface alloying was carried out by using TIG. As a side note, it is important that the powder mixture does not contain Iron. The steel substrate provided the Iron element during surface melting. An inverter TIG welding machine (Pars-Digital PSQ 250 AC/DC) was used with a constant voltage of 220 V an electrical current of 90, 110 and 130 A to heat and melt the pre-layer and substrate. The substrate was moved by a CNC table at a speed of 4.3 mm s−1. At the same time, Argon gas was used to protect the arc. The torch moved several times on all the points of part of the surface with an overlap of 50 %. The specimens after being cut, mounted and polished were characterized by optical microscopy (OM), a scanning electron microscope (SEM, JEOL JSM-6390), an energy dispersive X-ray spectroscope (EDS, Oxford Inca, Oxford Instruments) and X-ray diffraction (XRD, X'pert) to determine the present phases in the layer. Also, the hardness was measured by a hardness tester (Hysitron Inc., TriboScope® nanomechanical test instrument) in a Vickers unit with a load of 0.5 kg.

All electrochemical measurements were performed using the ivium vertex potentiostat in 1M HCl solution at ambient temperature after 30 min immersion. A conventional three-electrode cell was used with the bare and coated samples as the working electrodes, the platinum electrode as the counter electrode and the saturated calomel electrode as the reference electrode. It should be noted that all the reported potential in this text refers to SCE. The EIS measurements were carried out by impressing a 10 mV amplitude of ac signal and a wide frequency spectrum of 100 kHz–0.01 Hz on the OCP. Polarisation measurements were performed at a scan rate of 1 mV/s.

## 3 Results

### 3.1 The effect of electrical current

**Fig. 1:**
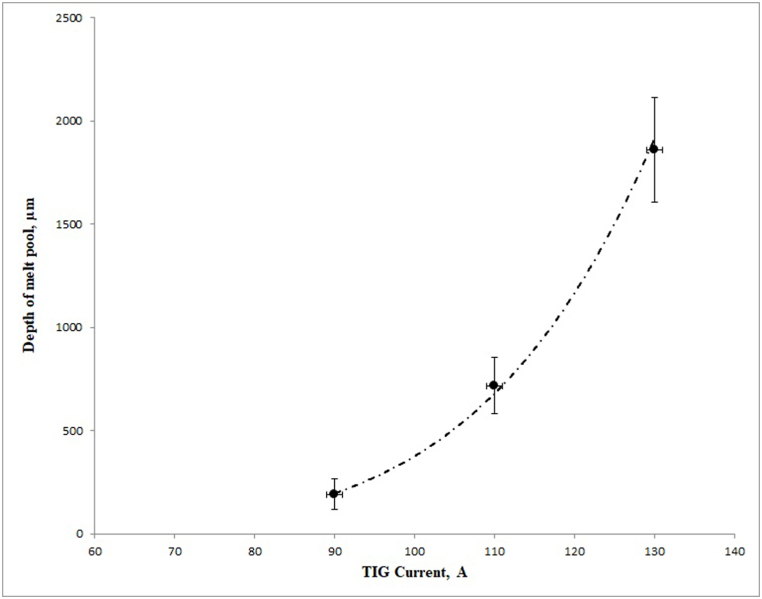
Influence of the TIG current on the depth of the melt pool.

**Fig. 2:**
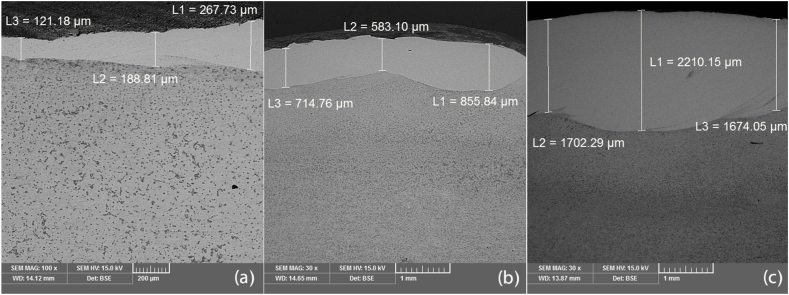
SEM images of the TIG current on the depth of the melt pool of (a) 90, (b) 110 and (c) 130 A.

Fig. 1 shows the effect of the applied current on the thickness of the layer. As shown in Fig. 1, the thickness of the layer increased with an increase in current. As the current increased from 90 to 130 A, the depth of the molten layer increased from 192 to 1862 μm. The change was considerable at the same time with the increase in current from 110 to 130 A, an increase in the depth of the layer was observed from 717 to 1862 μm. Fig. 2a-c shows the SEM images of the section of the substrate and layer at various electrical currents.

This thickness was not completely uniform. At various measures of current such as 90, 110, and 130 A, the average depths of the layers were about 192, 717, and 1862 μm, respectively.

### 3.2 Phases, chemical composition and microstructure

**Fig. 3:**
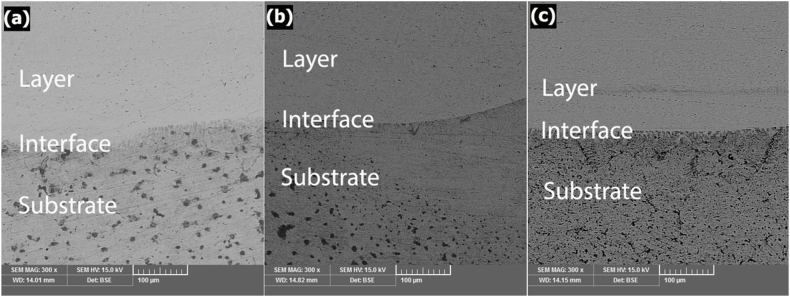
SEM microstructure of the cross-section of the coating/substrate at TIG current of (a) 90, (b) 110 and (c) 130 A.

**Fig. 4:**
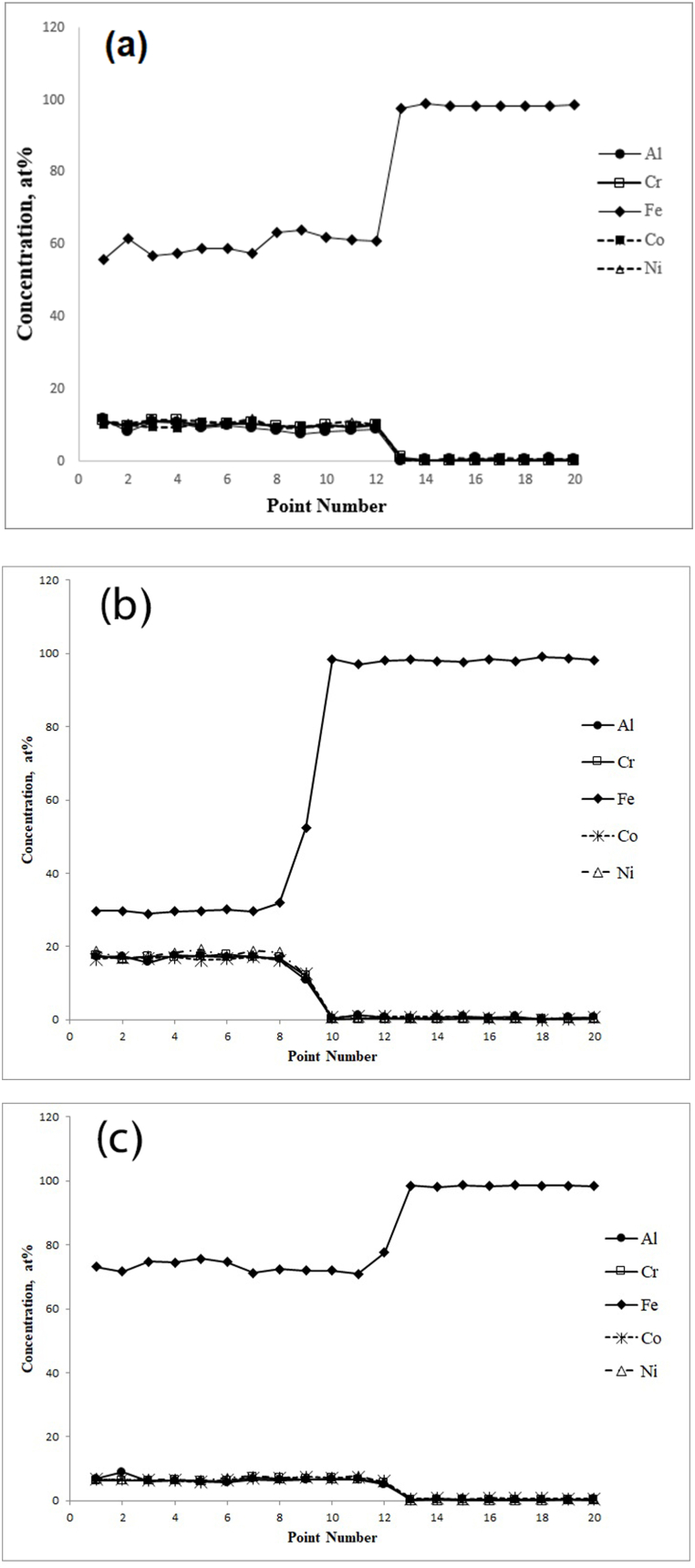
EDS of the cross-section of the coating produced using TIG current of (a) 90, (b) 110 and (c) 130 A.

The SEM (BSE) images of the layer specimens (Fig. 3(a–c)) show the various phases that were formed in the layers. Layer, interface, and substrate are clear in these images. The chemical composition gradient in the coatings was also measured using the EDS line scans (Fig. 4).

**Fig. 5:**
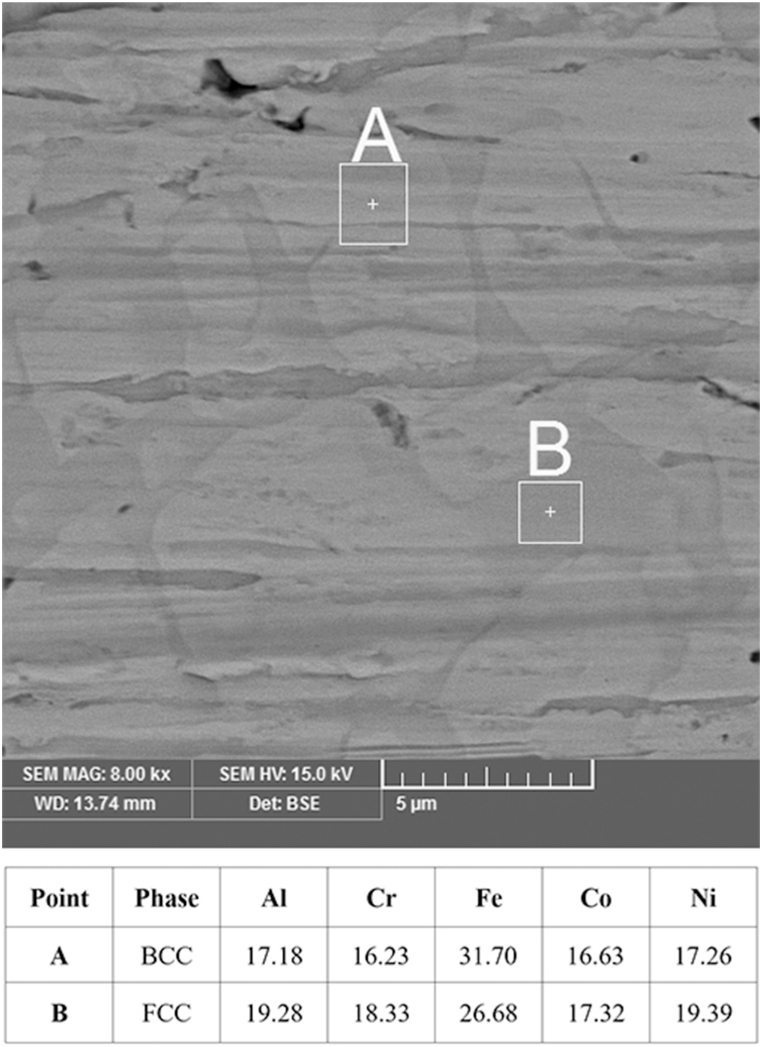
BSE micrographs obtained from the cross-section of the coatings produced at a TIG current of 110 A.

**Fig. 6:**
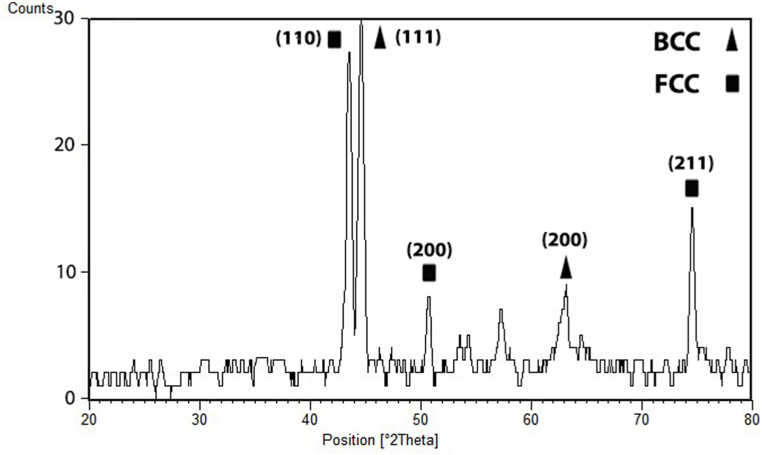
XRD spectra obtained from the coating that was produced using a TIG current of 110 A.

**Fig. 7:**
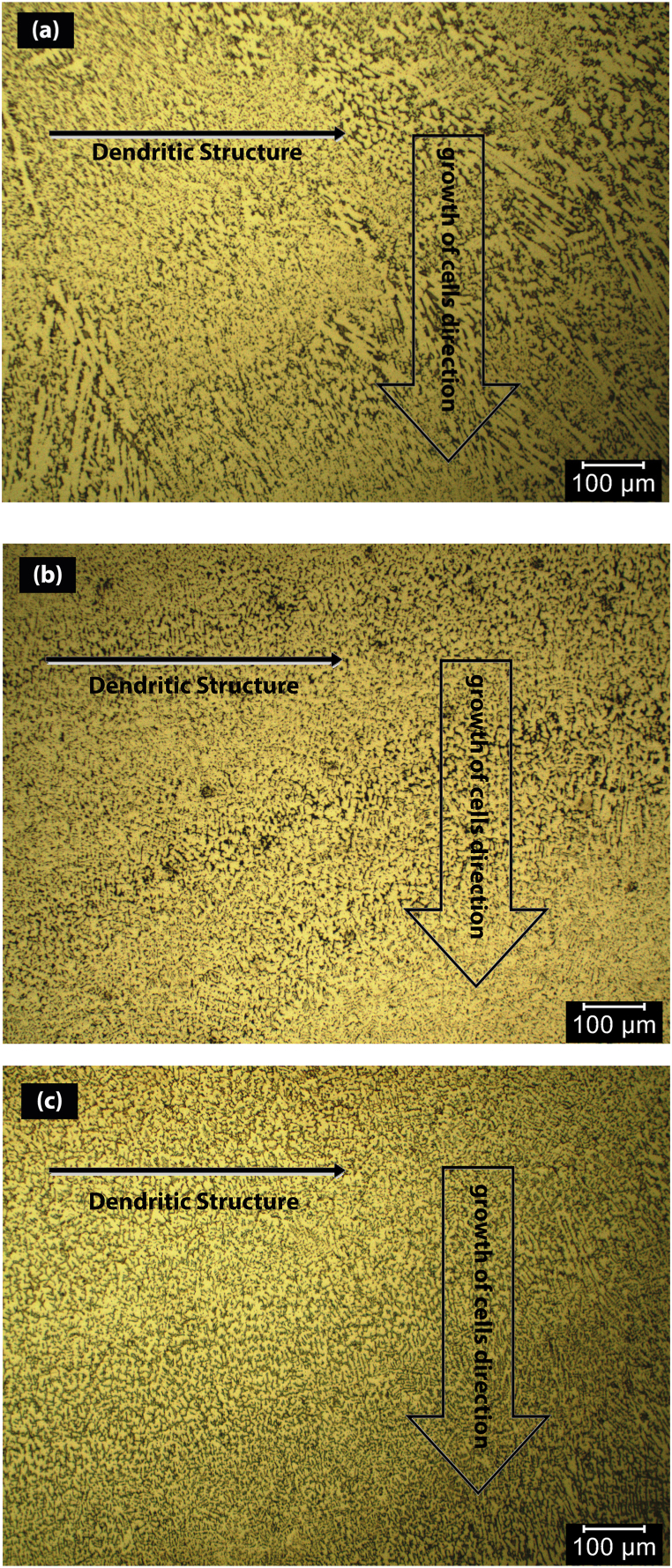
OM microstructure of the cross-section of the coating/substrate at TIG currents of (a) 90, (b) 110 and (c) 130 A.

At 90 A (Fig. 4a), the points showed about 9 at% Al, 10 at% Cr, 10 at% Co, 11 at% Ni and 60 at% Fe. At 110 A (Fig. 4b), the concentration of Fe was decreased, and the concentration of other elements was increased. The points displayed about 17 at% Al, 18 at% Cr, 16 at% Co, 17 at% Ni and 32 at% Fe. Unlike 110 A, at 130 A (Fig. 4c), with the increase of the electrical current, the concentration of Fe increased while the concentration of other elements decreased. The points displayed about 6 at% Al, 6 at% Cr, 7 at% Co, 7 at% Ni and 74 at% Fe. Finally, with regard to high entropy alloy definition, the results showed that high entropy alloy coating was produced at the electrical current of 110 A. SEM image of the coatings obtained at 110 A (Fig. 5) determined the stable phases in the coating. Their chemical compositions (characterised by EDS) are shown in Fig. 5. A phase (shown by letter A) in a matrix with another phase (shown by letter B) was observed at 110 A. The average concentration of Fe at points A and B was 32 and 27 at%, respectively. Fig. 6 represents the XRD results that were obtained from the layer formed using an electrical current of 110 A. The results illustrated that at the current of 110 A, the surface layer included BCC and FCC phase structures. One of the main problems in this kind of coating is the possibility of the formation of intermetallic compounds. But a significant presence of intermetallic compounds was not observed in samples. The optical microscope (OM) images of the layer at different electrical currents are displayed in Fig. 7a-c, which indicate a dendritic structure throughout the entire coating under different conditions. Also, no voids or cracks were observed in the entire coating.

### 3.3 Microhardness

**Fig. 8:**
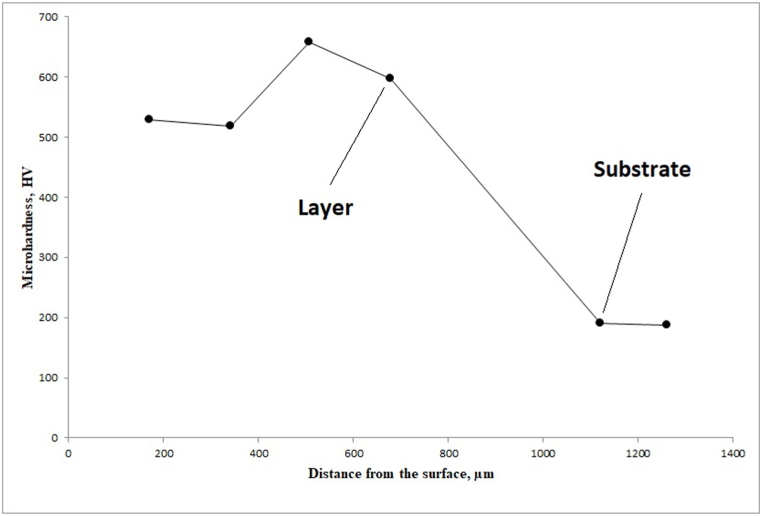
Average micro-hardness of the cross-section of the coating/substrate produced using a TIG current of 110 A.

Fig. 8 shows the average microhardness of the surfaces of the layer that were formed at the electrical current of 110 A. The layer coated by 110 A current had a microhardness of about 518–658 HV at the surface. There was a significant difference in microhardness between the coating and substrate. In all tests, the hardness of the substrate was in the range of 180–190 HV.

### 3.4 Electrochemical measurements

**Fig. 9:**
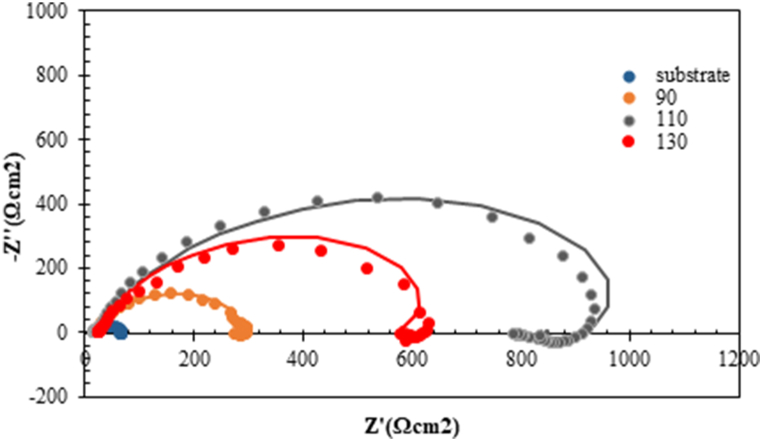
EIS Nyquist curves recorded on the bare and coated samples in 1M HCl solution at ambient temperature.

**Fig. 10:**
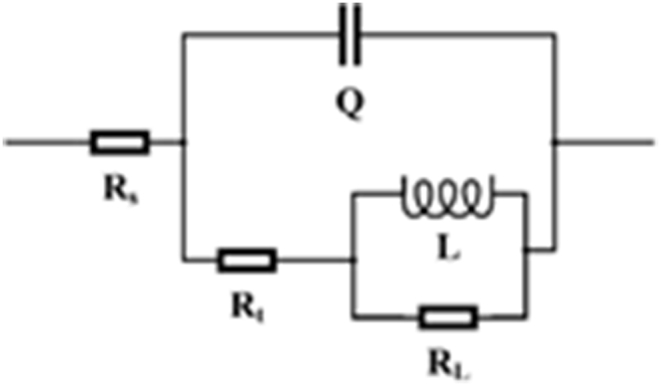
Equivalent circuit model for impedance data fitting.

In order to investigate the corrosion behaviour of the samples, an electrochemical impedance test was performed on the samples. Electrochemical impedance is regarded as one of the practice tests to evaluate the behaviour of the interface between coatings and solution. The Nyquist plots for the corrosion of bare and coated samples in 1M HCl at ambient temperature are depicted in Fig. 9. As can be seen, in the HCl solution, all electrochemical Nyquist curves had the same shape, such that the compact semicircle could be clearly seen. Fig. 9 also displays that the diameters of the electrochemical Nyquist curves were visibly different from each other. The analysis of these curves was performed based on the equivalent circuit, as shown in Fig. 10. In this circuit, R-Q is attributed to the charge transfer reaction; it has been replaced by the constant phase element due to the non-ideal dual-layer capacitor. R_L_ is the resistance of the induction process and L represents pseudo-inductance. The results of this analysis are presented in Table 1.

**Table 1:** The result of Nyquist plots for the corrosion of bare and coated samples in 1M HCl.

TIG Current	Rs	Rp	CPE	n	R_L_	L (Hcm2)
	(Ωcm^2^)	(Ωcm^2^)	(μSs^n^cm^-2^)		(Ωcm^2^)	
substrate	10.5	62.5	545.3	0.84	4.35	9.77
90	20.5	271.1	68.2	0.85	44.16	1.95
110	16.3	834.2	23.7	0.77	419.6	13.56
130	22.9	566.2	45.7	0.82	290.7	8.17

**Fig. 11:**
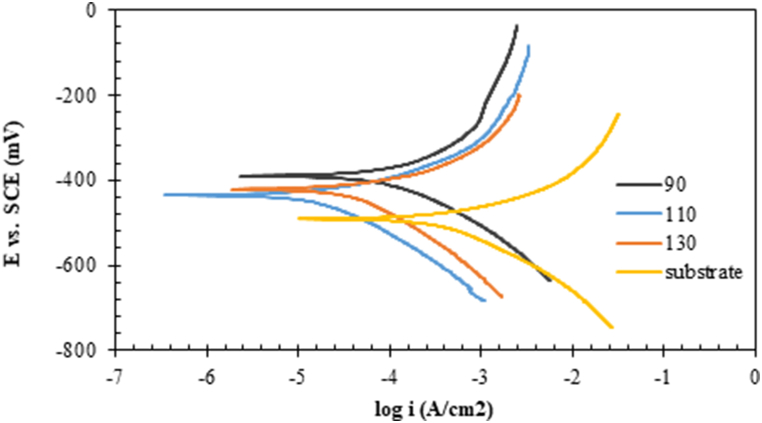
Potentiodynamic polarisation curves of bare and coated samples in 1M HCl at ambient temperature.

In order to more accurately investigate the electrochemical properties of the coatings on the substrate, an electrochemical polarisation test was performed on the samples. Fig. 11 shows the results of this test in 1M HCl solution. As can be seen, by applying the coating to the steel specimen, the polarisation curves were shifted to the left and the lower current densities. On the other hand, by increasing the current in the TIG process from 90 to 110 A, the polarisation curve first moved to the left; with a further increase in current, it moved slightly to the right. As shown in Fig. 11, in the HCl solution, the passive layer was not formed even on the 110 A sample. Table 2 shows the results of the analysis of electrochemical polarisation curves.

**Table 2:** The results of the analysis of electrochemical polarisation curves in HCl solution.

sample	i_corr_ (μA/cm^2^)	E_corr_ vs. SCE (mV)
substrate	572.2	−489.5
90	127.2	−407
110	39.7	−450
130	63.6	−429

**Fig. 12:**
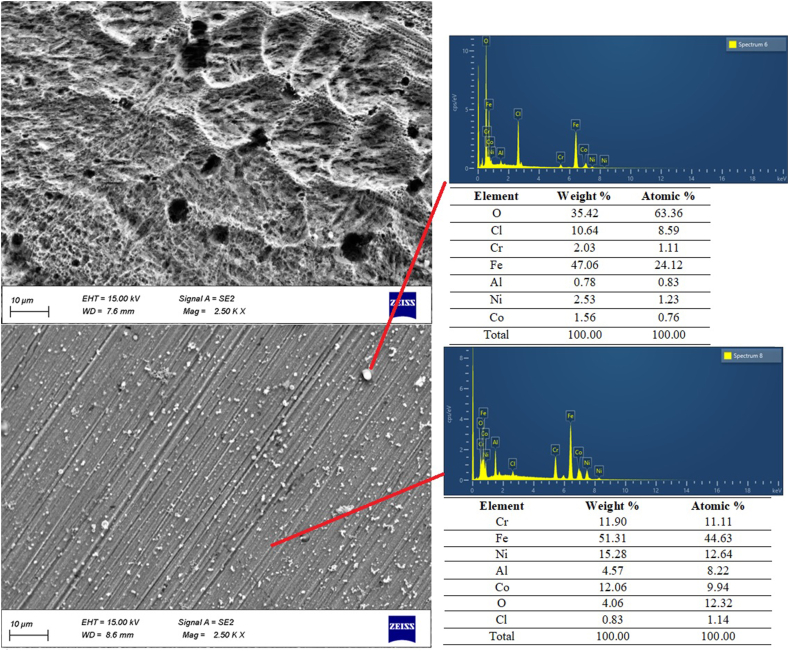
SEM images and EDS analysis of substrate and 110 A samples after 48 h immersion in HCl solution.

Surface features of the substrate and 110 A samples were examined under SEM after 48 h of immersion in HCl solution. Fig. 12 shows SEM images of substrate and 110A coated samples.

## 4 Discussion

### 4.1 The effect of electrical current

The increase in the electrical current in the TIG welding process would lead to an increase in heat input, thus, increasing the depth of the molten pool. The relationship between the depth of the molten pool and the electrical current is not linear, because Eq. (1) [18] shows the correlation between the parameters of welding voltage (E), electric current (I), electrode movement speed (V) and thermal efficiency (η) with the heat input (Q). In this case, the heat is directly related to the input current and voltage, and the increase in current causes a simultaneous increase in the voltage. In other words, in addition to directly increasing the heat, the current also increases the heat by increasing the voltage. Therefore, the increase in the depth of the molten pool was significantly greater from 110 A to 130 A compared to 90 A–110 A (Fig. 1).

(1) Q = η × E × I

### 4.2 Phases, chemical composition and microstructure

The relationship between the electric current and the depth of the melted layer is significant because it determines the chemical composition of the coating, especially the part of the coat composition provided by the substrate iron which melts along with the pre-coat. Also, the increase in the depth of the molten pool results in a longer solidification time, which allows time for the distribution of the alloy elements. Therefore, the forces applied to the molten weld pool in the TIG welding process, such as Buoyant forces, the Lorentz force, and Shear stress [14] are increased, and there is sufficient time to apply them. As a result, the liquid mixing will be improved reasonably in the molten weld pool to provide the necessary conditions for creating a uniform layer. The volume of the molten weld pool plays a critical role in creating a desirable layer, especially for high entropy coatings. This is because if the molten pool is shallow the amount of iron entering the pool will not be sufficient, and the iron required to create the high entropy alloy, which should be at least 5 at%, will not be available. On the other hand, if the electric current (and consequently the depth) of the molten pool is very high, the amount of iron entering the molten pool exceeds the maximum permissible value of 35 at% and does not meet the requirement for a high entropy alloy. Therefore, it is essential to achieve optimum electric current. Contrary to the previous supposition that formation of multiple intermetallic compounds, as suggested by statistical thermodynamics and also the Boltzmann equation, indicates that increasing the number of elements in an alloy reduces the possibility of solid solution formation [30], it is stated that as the number of elements increases, the entropy of the system also increases. This reduction in Gibbs free energy (ΔG) variation enhances the possibility of solid solution formation. Accordingly, another requirement for the formation of high entropy alloys is the presence of at least five elements in the compound. A comparison of these conditions with the actual governing conditions of the coatings shows that the layer created under the 90A current cannot be a high entropy alloy because its iron content is greater than 60 at% (Fig. 4a). However, contrary to the expectation that at 110 A with increased electric current and depth of the molten weld pool the iron content would increase, instead the iron content decreased to 32 at% and other elements were in the range of 16–17 at% (Fig. 4b). At 90 A, the force applied by the electric arc on the pre-coat was not probably sufficient and the powders compacted on the substrate moved forward, so that a small portion of the initial layer melted and the majority of the molten pool was formed from the iron of substrate. However, at 110 A the electric arc provided the force required to prevent the motion of the pre-shield, and in addition to the substrate, a majority of the pre-coat also melted and formed the compounds of the molten weld pool. By increasing the current to 130A and increasing the depth of the molten weld pool, the iron content increased to 73 at% (Fig. 4c) and exceeded the threshold determined for the high entropy alloys. The results of the XRD (Fig. 6) on the coat created by the 110A current showed that the coat consists of two-phase structures of FCC and BCC solid solutions, BCC being the matrix and FCC the second phase structure. The XRD analysis and calculation of Bragg's law reveal the presence of a BCC phase with a lattice constant of a = 0.286 nm and an FCC phase with a lattice constant of a = 0.357 nm. Cai et al.’s results [31] confirm obtained results because they calculated lattice constant for FCC and BCC 0.366 and 0.287, respectively. The SEM images in Fig. 5 are also in full agreement with the XRD results, as two distinct phases (one shown by A and the other by B) can be distinguished. As shown in Fig. 5, phase A has approximately 32 at% of iron and Phase B has approximately 27 at% of iron. The phase richer in iron has a BCC phase structure [31] while the phase with a low amount of iron has an FCC phase structure. The atomic percentage of iron in two mentioned phases is similar, which is why the color contrast between the two phases is not significantly different (Fig. 5). However, according to the evidence obtained and the findings of Cai et al., the FCC phase is stable for iron amounts below 30 atomic percent while the BCC phase is stable for higher amounts of iron. Both of them have five major elements, and all the elements have concentrations between 5 and 35 at%, and thus are high-entropy alloys. On the other hand, previous studies [32] show that the AlCrCoNiFe compound is prone to the formation of a solid solution. Also, in the calculations based on Eqs. (2), (3), (4)) [33] and the atomic percentages obtained from the shielding elements (Fig. 4b), the value of Ω the parameter introduced by Yang and Zhang [32,34,35] was 1.7, and the formation of the solid solution could be predicted from the diagrams presented by Yang et al. [32].

(2) ΔS_mix_ = RΣX_i_LnX_i_

(3) Ω=(T_m_.ΔS_mix_) / |ΔH_mix_|

(4) T_m_ = ΣX_i_ (T_m_)_i_

The main reason for the lack of intermetallic compounds is rapid solidification. Because different atomic radii in the HEA composition leads to the increase of the solid-liquid interface energy and the difficulty of the long-range diffusion of atoms in the crystal lattice, thus favouring the nucleation of solid solution and decreasing the growth rate of intermetallic compounds. Zhang et al. delicately studied the influences of laser rapid solidification on the microstructure and phase structure in the HEA coatings. They calculated the nucleation incubation time for various competing phases and indicated that the growth of intermetallic compounds will be hampered if the solidification rate is sufficiently high.

As shown in Fig. 7, the microstructure of the shield was completely dendritic. Similar microstructures have been observed in various studies [6,9,36]. The major determinants in the formation of microstructures by solidification are the temperature gradient ratio and growth rate [20]. In the molten pool resulting from the TIG process, with the development of solidification, the ratio of the gradient to the rate of formation is reduced and the cells are formed on the surface rather than deeper. The direction of cell growth is toward the substrate, which is influenced by the transfer of heat into the molten weld pool.

### 4.3 Microhardness

The hardness predicted in the studies [[37], [38], [39], [40], [41]] for the high-entropy AlCrCoNiFe alloy has been between 200 and 700 Vickers based on the exact chemical composition, production method and subsequent heat treatment. As shown in Fig. 8, the hardness of the layer is between 500 and 700 Vickers and is significantly different from the substrate due to the precipitation hardening and the presence of a second phase in the layer structure.

### 4.4 Electrochemical measurements

Compact semicircles in electrochemical Nyquist curves indicate that the electrochemical behaviour of the samples is controlled by the charge transfer process [42,43]. In addition to the compact semicircle at high frequencies, there is a small loop at the low frequencies. This semicircle, which is below the y-axis, can be the result of the release of the adsorbed ions on the surface and the separation of the corrosion products from the surface [44,45].

The diameter of Nyquist curves has a direct relation to corrosion resistance; so, the higher diameter of the curves indicates higher corrosion resistance [46]. According to Fig. 9, in the HCl solution, the diameter of the semicircle of the substrate was smaller than that of the coated samples, thus indicating improvement in corrosion resistance by creating a high entropy coating on the surface. The corrosion resistance of the samples can be predicted as follows:

Substrate< 90A < 130A < 110A

The polarisation resistance can be used to evaluate the corrosion resistance of the samples. The higher the value of Rct, the higher the density of protective coating, which means the better the effect of isolating corrosive medium. It is clear from Table 1 optimum coating (formed via 110 A) has a higher Rp value of 834 cm^2^ compared to the other HEA coating.

As shown in Table 1, by creating the coating on the substrate, the values of the constant phase element were decreased, thus indicating a decline in the access of corrosive ions to the substrate surface, and therefore, the corrosion process [47,48].

The lack of a passive layer in electrochemical polarisation curves can be explained through the reactions occurring on the surface. In fact, after placing the sample with the coating in the solution, an oxide film consisting of iron oxides, nickel oxide, aluminium oxide, cobalt oxide and chromium oxide was formed on the surface. However, in the acidic solution at 25 °C, the ΔG^0^ value related to the reaction of these oxides with hydrochloric acid was negative (except aluminium). For example, the reaction for nickel oxide is as follows:

NiO + 2HCl → NiCl_2_ + H_2_O, ΔG^0^ = −90 kJ/mol

Following this reaction, the oxide form of oxygen is replaced by a chlorine ion, leading to the formation of a metal chloride, which is a water-soluble compound [49].

According to the results of the polarisation test, by creating a coating with a current of 90 A, the corrosion current density of the steel substrate in the HCl solution was decreased from 572 to 127 μA/cm^2^; by increasing the current up to 110 A, the corrosion current density in the HCl solution was decreased to reach its minimum. Indicating that this coating provides the best corrosion protection in comparison to the other coatings and suggest a much slower dissolution of the 110A sample coating in 1M HCl solution than other samples. According to Table 2, by creating the coating, the corrosion potential was shifted to the positive values; from a thermodynamic point of view, this indicated a lower corrosion tendency for the substrate [[50], [51], [52], [53], [54], [55]].

According to Fig. 12, the SEM image of the substrate reveals a generously corroded surface and the major type of corrosion is mainly uniform corrosion. Coated substrate represents the less-corroded area with a relatively flat surface remaining and there is only some white phase on the surface; Chemical composition analysis was conducted by EDS. As presented in Fig. 12, five alloy elements are well distributed inside the coating, furthermore, the major element of the white phase is oxygen.

### 4.5 Comparison with other works

The comparative analysis indicates that the TIG process for AlNiCoCrFe coating on plain carbon steel offers favorable outcomes in terms of coating thickness and bond strength, making it a viable option for applications requiring robust surface protection. The TIG process resulted in a thicker coating about 700–800 μm compared to PVD [56] and plasma spraying [17,18] about 5–10 and 100–150 μm, respectively, which can enhance durability but may also affect thermal performance. About hardness, While PVD [56] exhibits superior hardness about 1200–1300 HV [17,18], the hardness of the TIG-coated samples about 600–700 HV is still adequate for many applications, providing a good balance between toughness and wear resistance and it is similar to plasma spraying and laser cladding methods. The bond strength achieved with the TIG method is competitive about 30–40 MPa, ensuring good adhesion to the substrate, which is crucial for performance in demanding environments. While, other current methods such as plasma spraying [18], PVD [56] and laser cladding [[7], [8], [9]] have an average bonding strength about 25, 15 and 35 MPa respectively.

## 5 Conclusions

(1) The TIG welding method was utilised successfully to form high entropy alloy coating of AlNiCoCrFe with high density and hardness on a steel substrate.
(2) The electrical current and depth of the melted layer produced during the surface treatment had a significant effect on the formation of a high entropy alloy.
(3) In this study, the optimum electrical current was 110 A, at which a high entropy alloy with BCC and FCC phase structures was produced. Lower currents could not escape the powders from the steel substrate surface. Higher currents also formed a layer with more depth, which resulted in an increase in the Fe concentration above the critical depth needed to produce a high entropy alloy.
(4) The layer coated had a microhardness of about 518–658 HV at the surface. There was a significant difference between the coating and substrate.
(5) Electrochemical results showed that coating prepared by 110A electrical current exhibited the optimum corrosion resistance in 1M HCl solution, which had higher corrosion potential, lower corrosion current density and higher charge-transfer resistance than the other coatings.

## CRediT authorship contribution statement

**Mahmoud Ardeshir:** Writing – original draft, Data curation. **Mardali Yousefpour:** Writing – review & editing, Supervision, Methodology, Funding acquisition, Data curation. **Seyad Mohammad Sadegh Nourbabksh:** Supervision. **Mansoor Bozorg:** Supervision.

## Data availability

Data will be made available on request.

## Submission declaration

• The work described has not been published previously except in the form of a preprint, an abstract, a published lecture or academic thesis. See our policy on multiple, redundant or concurrent publication.
• The article is not under consideration for publication elsewhere.
• The article's publication is approved by all authors and tacitly or explicitly by the responsible authorities where the work was carried out.
• Tf accepted, the article will not be published elsewhere in the same form, in English or in any other language, including electronically without the written consent of the copyright-holder.

## Declaration of competing interest

The authors declare that they have no known competing financial interests or personal relationships that could have appeared to influence the work reported in this paper.
